# Mosaicism for a pathogenic *MFN2* mutation causes minimal clinical features of CMT2A in the parent of a severely affected child

**DOI:** 10.1007/s10048-016-0504-2

**Published:** 2017-01-06

**Authors:** Katherine Schon, Olivera Spasic-Boskovic, Kim Brugger, Tracey D. Graves, Stephen Abbs, Soo-Mi Park, Gautam Ambegaonkar, Ruth Armstrong

**Affiliations:** 1 East Anglian Medical Genetics Service, Cambridge University Hospitals NHS Foundation Trust, Cambridge, CB2 0QQ UK; 2 Department of Neurology, Hinchingbrooke Hospital, Hinchingbrooke Park, Huntingdon, PE29 6NT UK; 3 Department of Paediatric Neurology, Cambridge University Hospitals NHS Foundation Trust, Cambridge, CB2 0QQ UK

**Keywords:** MFN2, CMT2A, Mosaicism, Charcot-Marie-tooth disease

## Abstract

Charcot-Marie-Tooth disease (CMT) refers to a genetically heterogeneous group of disorders which cause a peripheral motor and sensory neuropathy. The overall prevalence is 1 in 2500 individuals. Mutations in the *MFN2* gene are the commonest cause for the axonal (CMT2) type. We describe a Caucasian 5-year old girl affected by CMT2A since the age of 2 years. She presented with unsteady gait, in-turning of the feet and progressive foot deformities. Nerve conduction studies suggested an axonal neuropathy and molecular testing identified a previously reported pathogenic variant c.1090C > T, p.(Arg364Trp) in the *MFN2* gene. This variant was also detected in a mosaic state in blood and saliva by Sanger sequencing in her subjectively healthy father. Next generation sequencing showed that the level of mosaicism was 21% in blood and 24% in saliva. A high recurrence risk was given because the father had proven somatic mosaicism and an affected child implying gonadal mosaicism. The parents were referred for pre-implantation genetic diagnosis. To the best of our knowledge, this is the first reported case of somatic mosaicism for *MFN2*. This study has important implications for genetic counselling in families with CMT2A.

## Introduction

Charcot-Marie-Tooth (CMT) disease refers to a heterogeneous group of hereditary disorders which cause a peripheral motor and sensory neuropathy. The overall prevalence is approximately 1 in 2500 [1]. They are also genetically heterogeneous with over 60 reported genes [2]. Autosomal dominant, autosomal recessive and X-linked inheritance have been described. CMT can also be classified on the basis of nerve conduction velocity into demyelinating (CMT1) and axonal (CMT2) types.

Charcot-Marie-Tooth disease type 2A is caused by mutations in the *MFN2* (mitofusin 2) gene (OMIM 608507). Autosomal dominant inheritance or de novo dominant mutations are reported in most families [3, 4]. Autosomal recessive inheritance or semi-dominant inheritance has also been reported, mostly with a more severe phenotype [5, 6]. CMT2A caused by heterozygous mutations in *MFN2* has a variable phenotype with an early onset form with age at onset <10 years (mean age 3.5 years) and a late onset form with age at onset > = 10 years (mean age 20.5 years) [3]. The early onset form is more common and usually has a more severe phenotype [3]. CMT2A is characterised by earlier and more severe involvement of the lower extremities, more severe motor than sensory involvement and normal or slightly decreased nerve conduction velocities. Some patients have additional features of optic atrophy and/or spinal cord abnormalities [4].

Mosaicism occurs when an individual who has developed from a single fertilised egg has two or more cell lines which are discordant for a genetic feature [7]. This could be a chromosomal abnormality, for example in mosaic Trisomy 21, or it could be a sequence variant. The occurrence of families with more than one affected child for an apparently de novo dominant or X-linked condition (i.e. where the mutation was not detected on testing parental samples) can be explained by gonadal (or germline) mosaicism. Individuals may have gonadal mosaicism only, or may have somatic and gonadal mosaicism. Somatic mosaicism is being increasingly detected as a result of next generation sequencing [8].

## Materials and Methods

The family was ascertained through referral to the East Anglian Medical Genetics Service. Informed consent for publication was obtained. The proband and her father were assessed by the neurologists (GA and TDG). Electrophysiology studies (electromyography and nerve conduction studies) were performed in the proband. A peripheral blood DNA sample from the proband was tested for copy number changes on chromosome 17 using the Agilent ISCA 8x60K v2 array and CytoGenomics software, Edition 2.5.8.11 (Build 37). This showed she did not have the CMT1A duplication or hereditary neuropathy with liability to pressure palsies (HNPP) deletion. Next generation sequencing of the coding region (+/− 5 bp) was performed using the Illumina TruSight One sequencing panel for the following genes: AARS (NM_001605.2); ARHGEF10 (NM_014629.2); ATL1 (NM_015915.4); CTDP1 (NM_004715.4); DNM2 (NM_001005361.2); DNMT1 (NM_001130823.1); DST (NM_015548.4); DYNC1H1 (NM_001376.4); EGR2 (NM_000399.3); FAM134B (NM_001034850.2); FBLN5 (NM_006329.3); FGD4 (NM_139241.2); FIG4 (NM_014845.5); GAN (NM_022041.3); GARS (NM_002047.2); GDAP1 (NM_018972.2); GJB1 (NM_000166.5); HOXD10 (NM_002148.3); HSPB1 (NM_001540.3); HSPB8 (NM_014365.2); IKBKAP (NM_003640.3); KARS (NM_001130089.1); KIF1A (NM_001244008.1); KIF1B (NM_015074.3); LITAF (NM_004862.3); LMNA (NM_170707.3); LRSAM1 (NM_138361.5); MED25 (NM_030973.3); MFN2 (NM_014874.3); MPZ (NM_000530.6); MTMR14 (NM_001077525.2); MTMR2 (NM_016156.5); NDRG1 (NM_006096.3); NGF (NM_002506.2); NTRK1 (NM_002529.3); PMP22 (NM_000304.2); PRPS1 (NM_002764.3); PRX (NM_181882.2); RAB7A (NM_004637.5); REEP1 (NM_022912.2); SBF2 (NM_030962.3); SH3TC2 (NM_024577.3); SLC12A6 (NM_133647.1); SOX10 (NM_006941.3); SPTLC1 (NM_006415.2); SPTLC2 (NM_004863.3); TRPV4 (NM_021625.4); WNK1 (NM_001184985.1); YARS (NM_003680.3). 99.2% of the target sequence within this panel was sequenced to a depth of 20 fold or more, with analytical sensitivity of 98.3% - 100% (95% Confidence Intervals).

Sanger sequencing with fluorescence sequence analysis was performed in peripheral blood samples from both parents and a saliva sample from the proband’s father. Next generation sequence analysis of the PCR product containing the pathogenic variant was subsequently performed in the father to quantify the level of mosaicism.

## Clinical Report

A four-year old, first born girl of an unrelated Caucasian couple was referred with gait abnormalities and progressively worsening foot deformities. The pregnancy and birth history was normal. She crawled at 6 months and walked at 12 months but her gait remained unsteady with recurrent falls and trips, particularly in the mornings. Progressive in-turning of her left foot was noted from age two and a half years and she was referred for physiotherapy. Physiotherapy review at age 3 ½ years revealed that she was unable to run or walk long distances (necessitating wheelchair use) and could only get up off the floor with half-kneeling. Parents also gave a history of difficulty climbing stairs and recurrent leg pain. There were no concerns about her upper limb function or power and speech and language development was normal. There were no swallowing difficulties or salivary drooling. There was no history of neuro-regression or seizures and no concerns regarding hearing or vision.

Head circumference was 49.5 cm (50th centile). She was noted to be hypermobile with Beighton score 5/9 and significant in-rolling of her left and right feet (L> > R). There was good antigravity power in her upper and lower limbs. Deep tendon reflexes were present in upper and lower limbs. Gower’s manoeuvre was negative. She walked with dystonic posturing of both feet and could not jump high. There was no dysarthria and no dysmetria; cranial nerve examination was normal with no ophthalmoplegia or ptosis.

Blood tests showed normal CK, lactate and acylcarnitine. Nerve conduction studies at age 3 ½ showed absent sensory and motor responses in the legs (tibial, common peroneal and sural nerves tested). Electromyography revealed a reduced interference pattern with no spontaneous activity in the motor units tested (tibialis anterior), suggestive of a severe axonal neuronopathy However motor nerve conduction studies in upper limbs was normal (58.8 m/s) which also raised the possibility of a spinal cord pathology but the MRI spine was normal. MRI of the brain and X-ray of the lower legs and hips were also undertaken and were normal.

**Fig. 1:**
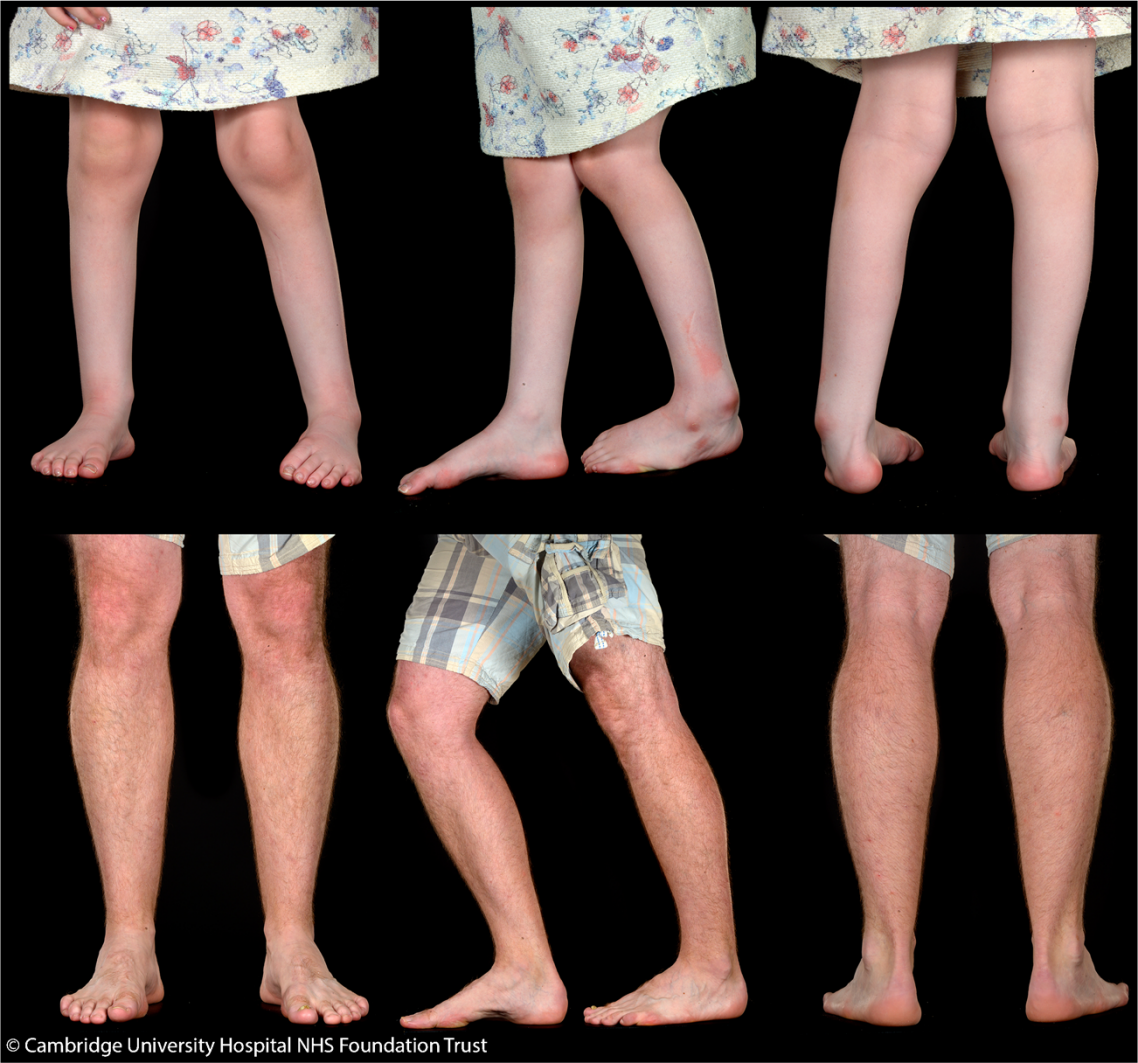
Clinical photographs showing muscle atrophy and foot deformities in the proband aged 5 years, and classical pes cavus with normal muscle bulk in her father aged 30 years

The in-turning of her left foot developed rapidly to a quite marked equinovalgus deformity with marked forefoot metatarsal plantar flexion. This was treated with ankle foot orthoses and serial castings. Figure 1 shows clinical photographs of her lower legs aged 5 years.

Genetic testing was performed for a panel of genes associated with CMT. A previously reported pathogenic variant [9, 10] was detected in the *MFN2* gene c.1090C > T, p.(Arg364Trp). This missense mutation affects a highly conserved amino acid and there is a moderate physicochemical difference between the arginine and tryptophan amino acids. Individuals with this mutation have been reported both with and without optic atrophy. No other variants were found. Subsequent ophthalmology review was normal and annual ophthalmology follow up was arranged.

**Fig. 2:**
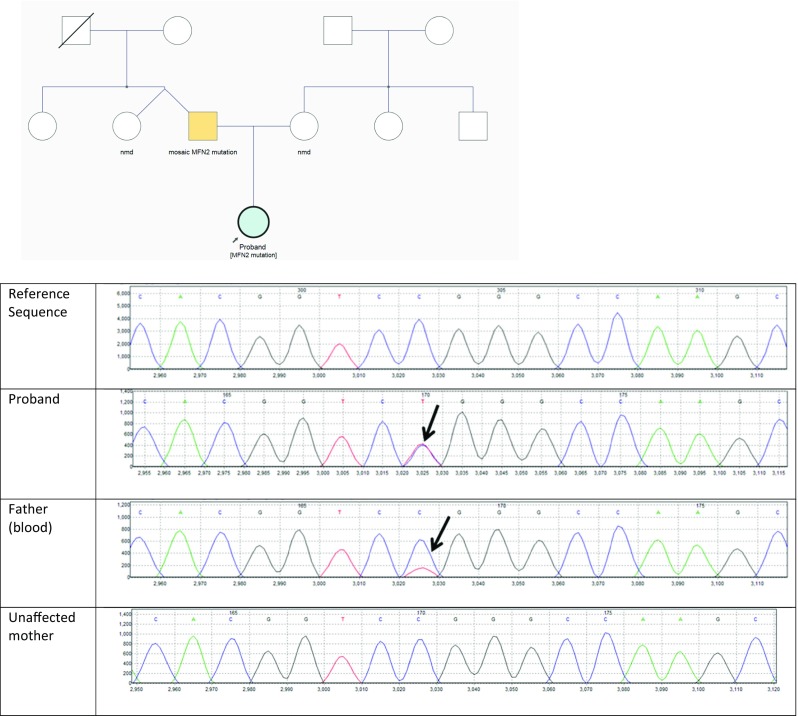
Pedigree and screenshot of sequencing data from Mutation Surveyor, showing the reference sequence, proband, father (peripheral blood) and mother. The c.1090C > T, p.(Arg364Trp) heterozygous variant is seen in the proband, and at a level of approximately 20% in her father

**Fig. 3:**
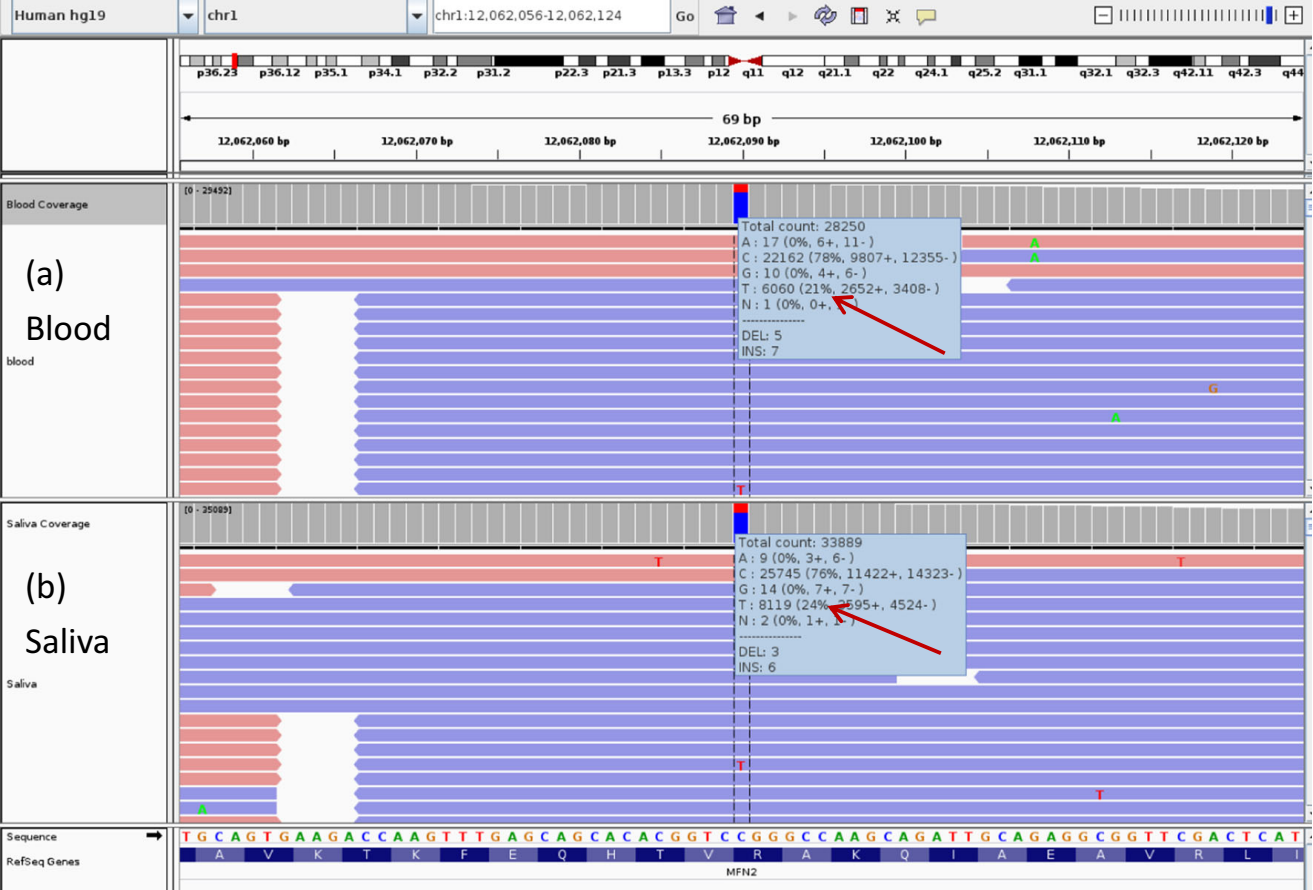
Integrative Genomics Viewer (IGV) screenshot of the c.1090C > T MFN2 pathogenic variant. (**a**) blood DNA sample and (**b**) saliva DNA sample from patient’s father. The level of mosaicism detected is 21% in the peripheral blood sample and 24% in the saliva sample

There was no reported family history of CMT. Both parents were keen runners. On initial examination, high arched feet were noted in her 30-year old father (see clinical photographs in Fig. 1). Testing of parental blood samples by fluorescent sequence analysis detected the familial *MFN2* mutation in her father at a low level of approximately 20% suggestive of mosaicism. Figure 2 shows the pedigree and Sanger sequencing data. The mutation was also detected in his saliva sample at a low level. Next generation sequence analysis was used to quantify the level of mosaicism and showed a level of 21% in blood and 24% in saliva. This is shown in Fig. 3.

On review, the father had normal motor milestones and had been a sporty child. He had been a member of the armed forces and enjoyed running as a hobby. On examination, he had classical pes cavus with callus formation on the outer aspect of the balls of his feet. He had no demonstrable weakness, reflexes were preserved. Sensory examination revealed reduced pinprick to mid-foot bilaterally and reduced vibration sense to the ankles. Ophthalmology review was normal.

The parents were advised that there was a high recurrence risk (up to 50%) in subsequent pregnancies. After discussion of the reproductive options, the parents were referred for pre-implantation genetic diagnosis.

## Discussion

Mutations in *MFN2* are the commonest genetic cause for axonal CMT. The proband showed typical features for the early onset form of the condition [3, 4]. Two recent studies of patients with CMT2A have revealed two patients with presumed gonadal mosaicism. Two siblings included in a recent study of 43 French patients with CMT2A were affected with early onset peripheral neuropathy and optic atrophy. A novel pathogenic missense variant (c.775C > T, p.(Arg259Cys) was detected in both siblings but not in their clinically unaffected parents [4]. A study of Czech hereditary motor and sensory neuropathy type II patients detected 8 pathogenic *MFN2* mutations, including two sisters affected with axonal CMT neuropathy. A novel pathogenic missense variant c.314C > G, p.(Thr105Arg) was detected in both sisters but not observed in their clinically unaffected parents [11]. To the best of our knowledge, somatic mosaicism has not been previously reported in CMT2A.

The proband’s mosaic father showed minimal clinical features of CMT. There is considerable variability is seen in dominantly inherited CMT2A, with some patients having a later onset, milder form of the disease [3, 4]. However, his very mild features are most likely to be explained by the somatic mosaicism.

Mosaicism has been previously reported in other forms of CMT, with a very variable clinical phenotype including asymptomatic with normal neurophysiology studies [12], subclinical disease [13], mild clinical disease [14–16] and typical presentations of CMT1A with somatic mosaicism found on genetic testing [17–19]. The mutations, level of mosaicism, clinical features and neurophysiology findings from the literature are summarised Table 1.

**Table 1:** Summary of mutations, level of mosaicism, clinical features and neurophysiology findings in individuals with mosaic CMT

**Reference**	**Gene**	**Mutation**	**% mosaicism**	**Reason for ascertainment**	**Clinical Features**	**Neurophysiology**
This family	*MFN2*	c.1090C > T, p.(Arg364Trp).	21% (blood) 24% (saliva)	Affected child	Bilateral pes cavus and reduced sensation in feet aged 30	Not done
Sorour	*PMP22*	Duplication	47.6% (blood)	Affected child	Clawing of toes and clumsiness since childhood, unsteadiness of gait from 40 years, difficulty with coordination in hands from 50 years	Normal median motor conduction velocity Significantly slow sural sensory conduction velocity
Liehr	*PMP22*	Duplication	40–58% (blood) 66% (hair root cells) 74% (nervous tissues) 51% (buccal mucosa)	Clinical CMT	Bilateral pes cavus, mild distal weakness of arms and legs, absent reflexes, sensory disturbance distal to elbow and knee at age 25	Motor NCV from median, ulnar and tibial nerves 18-20 m/s No sensory action potential could be recorded from sural, radial or median nerves
Rautenstrauss	*PMP22*	Duplication	60% blood 88% nerve tissue 72% muscle	Clinical CMT	Signs and symptoms of a demyelinating neuropathy aged 4	
Grehl	*PMP22*	Duplication	49% blood 74% sural nerve	Clinical CMT	Generalised weakness arms and legs from age 21, burning pains in shoulders, hands and feet, bilateral pes cavus, mild distally pronounced weakness in arms and legs, sensory disturbance distal to elbow and knee	Motor NCV from median, ulnar and tibial nerves 18-20 m/s No sensory action potential could be recorded from sural, radial or median nerves
Taioli	*PMP22*	c.117G > C, p.(Trp39Cys)	20% (blood)	2 affected children	Subclinical age 29 Pes cavus, decreased ankle jerk reflexes and vibration sensation in legs	Slight reduction in sural nerve action potentials
Borgulova	*GJB1*	c.784_786delTA	25% (blood)	Affected daughter	Asymptomatic Abnormal electrophysiology	Motor NCV from median 43.9 m/s, ulnar 52.9, tibial 33.3.
Kochanski	*GJB1*	p. Glu208Lys	Not stated	Affected grandson	Mild clinical CMT Unable to walk on heels from age 11, slowly progressive mild disease	Not done
Baker	*GJB1*	c.95G > A, p.(Arg32Lys)	Approx one third (blood)	Symptoms of carpal tunnel syndrome	Bilateral carpal tunnel syndrome age 39 Absent ankle jerk reflexes Mild stocking distribution pin hypoaesthesia	Ulnar, deep peroneal, tibial motor NCS normal, absent superficial peroneal SNAP, low amplitude sural SNAP
Fabrizi	*MPZ*	c.308G > A, p.(Gly74Glu)	20% (blood) 30% (skin, buccal epithelium, hair)	2 affected children	Asymptomatic. Pes planus.	Normal

Individuals with mild or subclinical CMT due to somatic mosaicism who have been ascertained through an affected child (or grandchild) have been described for CMT1A [16] , CMTX1 [13, 14], and CMT1E [15].

A family with CMT1B has been described with two severely affected sisters. A mutation in *MPZ* was found in both affected sisters and in mosaic form in their mother (20% in blood, 30% in skin, buccal epithelium and hair) who was clinically unaffected with normal neurophysiology studies [12].

Three cases of somatic mosaicism for the *PMP22* duplication with clinical findings typical for CMT1 have been described [16–18]. The level of mosaicism was fairly high in these individuals (range 40–88%). A man with mosaic CMTX1 has also been described who presented with symptoms of bilateral carpel tunnel syndrome [20].

The level of mosaicism detected in blood and saliva in the proband’s father was quite low (at around 20%). The similar levels of mosaicism found in blood and saliva suggest that the mosaic mutation happened early in embryogenesis since it is found in both ectodermal and mesodermal tissue. However, a nerve biopsy was not undertaken, so it is possible that the level of mosaicism in the neurons and/or Schwann cells may be higher. This could explain why he has foot deformities despite the low level of mosaicism. It has been recognised that it is difficult to predict disease severity in mosaic disorders [8].

The family which we have described highlights the need for parental testing in order to give reliable information about recurrence risk. The diagnosis of mosaic CMT2A would have been missed on the basis of family history without molecular testing. As the father has proven somatic mosaicism and an affected child, gonadal mosaicism is assumed. Estimating the recurrence risk in this situation is challenging. The family were counselled for a high risk of ‘up to 50%’ risk.

Next generation sequencing is likely to detect lower levels of mosaicism. It has been suggested that deep sequencing of blood for pathogenic apparently de novo mutations seen in children could be used to pick up low level mosaicism [21]. Families with no evidence of mosaicism would have a < 1% recurrence risk, but those with low level mosaicism would have a significantly higher risk.

Next generation sequencing was used to quantify the level of mosaicism in the father’s blood and saliva samples with greater accuracy than estimating the level from Sanger sequencing. However, we do not yet have a way of estimating recurrence risks based on the observed level of mosaicism. Further studies are needed to determine the most effective ways of estimating the recurrence risk and communicating the information to families.
